# Medicalization of global health 4: the universal health coverage campaign and the medicalization of global health

**DOI:** 10.3402/gha.v7.24004

**Published:** 2014-05-16

**Authors:** Jocalyn Clark

**Affiliations:** 1 icddr,b, Dhaka, Bangladesh; 2 Department of Medicine, University of Toronto, Toronto, Canada

**Keywords:** global health, universal health coverage, health care, medicalization, sociology

## Abstract

Universal health coverage (UHC) has emerged as the leading and recommended overarching health goal on the post-2015 development agenda, and is promoted with fervour. UHC has the backing of major medical and health institutions, and is designed to provide patients with universal access to needed health services without financial hardship, but is also projected to have ‘a transformative effect on poverty, hunger, and disease’. Multiple reports and resolutions support UHC and few offer critical analyses; but among these are concerns with imprecise definitions and the ability to implement UHC at the country level. A medicalization lens enriches these early critiques and identifies concerns that the UHC campaign contributes to the medicalization of global health. UHC conflates health with health care, thus assigning undue importance to (biomedical) health services and downgrading the social and structural determinants of health. There is poor evidence that UHC or health care alone improves population health outcomes, and in fact health care may worsen inequities. UHC is reductionistic because it focuses on preventative and curative actions delivered at the individual level, and ignores the social and political determinants of health and right to health that have been supported by decades of international work and commitments. UHC risks commodifying health care, which threatens the underlying principles of UHC of equity in access and of health care as a collective good.

Universal health coverage (UHC) – universal access to needed health services without financial hardship in paying for them (1) – has become the rallying cry for many in the global health community as the end of the Millennium Development Goals (MDG) programme approaches and a new agenda is being fashioned for 2015 and beyond. In the post-2015 ‘fervour’ (2), UHC has emerged as the leading recommended overarching health goal, and has the support and endorsement of major institutions and organisations like the World Health Organization (WHO), World Bank, the Foreign Policy and Global Health group, and The Rockefeller Foundation, among others, and a multitude of mostly supportive articles and editorials have been published in major medical journals, where UHC has been described by one leading journal as ‘an insuppressible right’ (3). Concerned with rising and catastrophic health care costs, especially in low- and middle-income countries (LMICs), more than 90 countries in December 2012 endorsed a United Nations resolution on UHC (4), which ensures its consideration in the broader development discussions. Described as no less than a ‘third global health transition’ (5) and an ‘epic transition’ (6), others have projected that UHC will not only improve access to health care but ‘could have a transformative effect in the battle against poverty, hunger, and disease’ (7). While it is indeed still an ‘aspirational slogan’ (8), it is also difficult to argue with a concept that offers the promise of health services for everyone in the world who needs them.

But in the zeal to embrace ‘the UHC mantra’ (9), the conceptualisation and consequences of UHC – not as a normative good but as *the* singular priority in global health – has inspired relatively little critical examination. Among the few published critiques, some have raised concerns about the imprecision attached to calls for UHC where, for example, the range, quality, and equity of services are inconsistently included and articulated in definitions (8–11), and this imprecision is said to threaten consensus and appropriate policy responses (8). Most reports and resolutions, in fact, fail to specify or make commitments to ensuring actual use, acceptability, quality, or the necessity or appropriateness of health services, all of which are necessary for UHC to be effective (8, 11). Others highlight the not inconsiderable challenges of implementing UHC at the country level, where the necessary financial, legal, and policy ingredients to support an effective health system and requisite human resources are often missing or inadequate (10, 12).

Insights from the tradition of medicalization analyses, which have roots in sociology and social constructionist approaches, enrich these critiques and allow for an examination of the UHC campaign and its framing of this singular global health priority. As I have described in-depth in Paper 1 of this series (13), and set out in a Box in the Paper 2 (14), a medicalization lens can be useful for critically examining the contemporary global health agenda, including what and how issues and problems get prioritised and framed, and what solutions are advanced. Unlike the previous cases of the medicalization of global health that I examine – global mental health movement (14) and the noncommunicable diseases agenda (15) – where a health condition is promoted, defined, and ‘treated’ in medicalized ways, here the interest is in how the UHC campaign positions access to health care as a core global health problem. Viewed through a medicalization lens, it appears that the UHC campaign is currently defined and framed in narrow terms, elevating the role of health care, individualising health by focusing on access to personal health services, and creating opportunities for commodification; together this reductionism risks medicalizing a key and high-profile component of the current global health agenda.

I trace three features.

## Conflating health care with health

First, too often in the current UHC discourse there is conflation of health care with health, an important and misleading conversion. A recent call to action in *The Lancet*, for example, interchangeably uses health and health care, asserting that UHC addresses existing constraints to ‘scaling up access to health’ and that the hope is to ensure ‘all of the world's people will have access to health at an affordable cost’ (7). Even the WHO director general's comments conflate health with health-care services when declaring in 2012 UHC to be ‘the single most powerful concept that public health has to offer’ (16). In fact, public health's main contribution and world view are to *deemphasise* the role of health-care services and instead highlight the multiple influences that act on health, in particular, the social and political determinants (17).

Positioning health care as equivalent to health in these development debates massively elevates the role of health care in alleviating global health problems that in reality are so centrally linked to poverty, power, and inequity. In turn, by positioning the primary solution to be health care – of which biomedicine is the dominant, modern practice – the UHC campaign medicalizes the agenda. As has been argued in other medicalization analyses where health care is offered as the solution to public health problems, the tendency is to focus strategies on increasing access to personal health services, often financial access. Policy responses then become about improving access to health care, displacing the more salient goal of improving health, and ignoring the socioeconomic conditions that created the vulnerability in the first place (18). Similarly, when it comes to redressing disparities, a medicalized view conflates health status disparities with health access disparities, overvaluing and overemphasising health-care access (18), or as Clift argues about UHC (9), assigning ‘undue importance to the delivery of health care services as a determinant of health outcomes and downgrading the importance of the [needed] economic, social, and environmental changes’. As decades of reports and commitments by WHO and others have stressed, there are a multitude of factors affecting health, and health care is only one of them (9, 19). Access to care is a necessary but not a sufficient determinant of health. Indeed, as O'Connell and colleagues point out, the definition of health present in the UN resolution on UHC is much broader than could be achieved through provision of basic or essential health services (8). And although Marmot was referring specifically to primary health care in relation to UHC, his caution applies more widely: improvement of access to care ‘is a worthy and necessary goal but, by itself, will not revolutionise global health, nor reduce large health inequalities’ (20).

This focus of the UHC campaign is of particular concern because the evidence suggests that health care on its own does not directly improve health outcomes. Historically, improvements in health and life expectancy were not the result of biomedicine but rather living standards, especially nutrition (21), and more contemporarily, even with substantial modern medical and technological advances, estimates range from 10% (17, 22) to 43% (23) of health being a consequence of health care. Broader social and economic improvements are more likely to produce health gains, and furthermore health care can cause harm, be wasteful, and be costly; Benatar stresses that the increasing medicalization of health generates unsustainable costs for societies (24). In relation to the UHC agenda, in particular, Clift argues ‘even if we achieve UHC as defined, … this would not necessarily produce the best outcomes’ (9) and Victora (19) warns that ‘it is possible to have UHC while still having poor and declining population health’. Moreno-Serra and Smith, in a comprehensive review, show the mixed and patchy evidence base to support the contention that UHC leads to better population health outcomes, reporting that the relationship depends a great deal on the setting and its quality of governance and institutions, the characteristics of the system, the specific health indicators measured, methodological limits, and the availability of data; they also highlight the importance of targeting care to those who need it (25). While they conclude, optimistically, that policy-makers should feel secure that steps toward universal coverage are an important strategy for population health, especially among poor people, this seems over optimistic: it is clear that there is a scarce evidence base to support causal claims (12). Indeed the 2013 World Health Report's central thesis is the strong need for more evidence on UHC (26).

## Reductionism in the UHC campaign

Second, the UHC goal risks medicalizing health because it is reductionistic. Even in its broadest definition that might include preventative, rehabilitative, and palliative care in addition to treatment, UHC clearly excludes the social and political determinants of health (20). As the 1978 Alma Ata Declaration first committed to (27) and the 2008 Commission on Social Determinants of Health affirmed (28), action on the social determinants of health is essential to alleviate the gross inequalities in health that exist around the world. In fact, several inputs to the post-2015 health and development agendas, including the Rio +20 declaration (29) and the inclusive World We Want consultation (30), emphasise the need for health goals that address these broader determinants, including environmental and living conditions, nutrition, income, education, gender, and race. Others broaden the frame (31, 32) to highlight the political determinants of health, arguing that the concentration of power in health agenda-setting, neoliberal ideologies, and other dimensions of politics can have an enormous impact on the health and inequalities of societies, and as such the political context must be accounted for. In contrast, UHC, however essential, ‘reflects preventive and curative actions delivered at (the) individual level’ (19).

In fact, positioning UHC with its individualistic approach as the preeminent health goal would appear to contradict previous global health commitments and resolutions that emphasise action on the social determinants of health and the need for political action and a long-term view. Without such a view, short-term thinking ‘incentivises a focus on interventions, physical entities (vaccines, medicines, devices, equipment) that one can buy, distribute, and count the effects of quickly’ (33). UHC has been described as a ‘silver bullet solution’ to health-care needs in LMICs (12), and when focused on expanding networks of health-care professionals and institutions, can produce a medicalized vision of health that simply makes more medical treatments available to more people (24). Like other target and priority-settings in global health, silver or magic bullet solutions are often favoured because they are seen as being easier to monitor, implement, and measure; able to produce immediate results and quick wins; and aligned with the existing agendas of national or international institutions (34, 35). But emphasis on the individual level deflects attention from the political context and from population level interventions such as taxation of harmful products; legislation of nutritional content of foods; and policy reforms to promote physical activity, fund pre-school education, improve housing quality or ensure water and sanitation infrastructure, among others, that can improve communities’ health (19, 20). Ignoring the social and political context means that the UHC goal can overlook the action needed outside the health sector, including the economic and social sectors enshrined in Alma Ata as of ‘basic importance to the fullest attainment of health for all’ (27). While some proponents claim that UHC is rooted in the human right to health (36), others argue it ignores international agreements to health and human rights (11, 37): Ooms and colleagues assert that UHC on its own is not enough to ensure the right to health without policy commitments to also ensure underlying determinants of health, and more robust civil society participation and collaboration with the communities whose health is at stake (2).

## Financing eclipses service delivery

The third issue fuelling the medicalization of health is the priority given in the UHC campaign to financing rather than service delivery. Such framing – where the promise is said to lie in pooled financing not pooled provision (12) – and in particular its lack of explicit support for public health systems strengthening, provides wide opportunities for profit-making and risks commodifying health care.

As Sengupta argues (12), the predominant definition of UHC is ‘a health financing system based on pooling of funds to provide health coverage for a country's entire population, often in the form of a ‘basic package’ of services made available through health insurance and provided by a growing private sector’. Most reports fail to emphasise the importance of public health services, instead focusing on cost-effectiveness and efficiency (12), which are values of medicalization (38). In the leading model of UHC, the state's role is to manage, regulate, and possibly purchase services, but the health services themselves are conceived as marketable commodities and they create an entry path for private insurance companies, private health-care providers, and managed care organisations (12). Thus, UHC can contribute to new and broader health-care enterprises by transforming the health-care needs of a population into specific commodities, defined by (mostly medical) experts, for economic markets (39). As with the growing commodification of aging (39), this is a form of medicalization. Such a model ignores the implications for equity, whereby private systems may avoid providing care to the poor, aged, chronically ill, or other patients who have conditions requiring costly or long-term care. In turn, this risks diverting attention and investment away from strengthening or rebuilding public health systems to provide UHC. Or, as Sengupta argues, this represents a ‘private sellout of health systems via UHC’ (12).

The focus in the current UHC campaign and this framing also represents a turn away from Alma Ata commitments and the thrust of most campaigns for health systems strengthening that seek integrated, equitable, and community-driven systems. Freedman and colleagues have proposed a conceptualisation of the health system as a core social institution that functions at the interface between individuals and wider structures of power (40, 41), which is thus capable of setting and reinforcing social norms. A context in which health care is bought and sold like a traded commodity risks the social legitimisation of the exclusion of those without the ability to pay. Indeed, even strong proponents of UHC suggest there is no guarantee that it can ensure that health care remains a collective good (7), and critics call instead for a strengthening of the underfinanced public sector (12, 42).

## How can the UHC campaign avoid medicalization?

There appears to be several ways that the UHC campaign can broaden the discourse to avoid contributing to or fuelling the medicalization of global health. First, it would seem essential to cease confounding health and health care, which is misleading and inaccurate. Irrespective of one's view of the relative contribution of health services or the health system to health, it is wrong to conflate in debates on UHC the terms health care (or health services) with health. This serves to erroneously promote the idea that health is achieved by health care alone, and elevates the position and role of biomedicine in solutions, policy responses, and investment, distorting the broader determinants of health and threatening the success of proposed interventions.

Prioritising a goal that emphasises the importance of access to health care takes attention away from the social and political determinants of health, which as a second recommendation, clearly must be better incorporated into the UHC campaign and goals. To galvanise support for this broadened campaign and goal, Marmot says that the global health community can take action on the social determinants through: ‘changes in clinical practice, partnership working, advocacy, education and training, and employment conditions of health-sector workers’ (20). Health workers can also lobby for a much broadened goal that avoids medicalization, perhaps along the lines of a recent Go4Health report that advocates for a broad goal of realising the right to health for everyone, supported by two secondary goals of UHC anchored in the right to health and in healthy social and physical environments (43). Further, Navarro and others argue that no consideration of the determinants of health is complete without examining the politics and power relations of the system in which priorities emerge (31, 35), and thus more attention to how the global political economy enables or obstructs achievement of UHC is needed.

Third, equity would seem a particularly important dimension to tackle further within UHC efforts as it appears to invoke a difference of opinion (26, 32, 44, 45). Whilst the early positioning of UHC would suggest that universalism and removing financial hardship equate with equity, some commentators argue that simply providing coverage of the status quo (more of the same) will reproduce present inequities (8, 32). Furthermore, health inequities can increase when, for example, only wage earners or those working in formal sectors access available health services, excluding the poor (44). In O'Connell and colleagues’ analysis (8), the assumption that equity is a natural consequence of UHC contrasts with the evidence showing that improved equity is ‘conditional on how UHC terms and policies are defined, designed, implemented, and sequenced’. Consequently, an explicit equity focus will need ‘hard-wired’ measurement and independent accountability, which has been highlighted in critical analyses of the MDG targets and implementation (32, 34); otherwise, the UHC campaigns risks worsening inequities. As Waage and colleagues (34) argue more generally but relevantly for extending coverage of universal health care, ‘issues of equity arise because many goals target attainment of a specific minimum standard … [but] to bring people above this threshold might mean a focus on those for whom least effort is required, neglecting groups that, for geographical, ethnic, or other reasons, are more difficult to reach, thereby increasing inequity’.

Fourth, debates about the value and implementation of UHC must further incorporate discussions and analyses of the political dimensions (as mentioned earlier), and must specify the roles and appropriate contributions of both the public and private sectors, recognising the power of vested interests. The normative analytical frameworks of global health research (biostatistics, epidemiology, health economics) concern themselves with more proximate indicators of health, excluding due attention to the root causes and to the politics in public health, a scenario that Navarro describes as disinclining researchers to tackle the ‘dirty issues’ that may disrupt the neutral agendas of public funders (31, 46). But more analysis of the political dimensions and the global political economy is very much needed, and would widen the discourse beyond its current framing as a largely technical and financing issue; indeed, as Stuckler and colleagues have argued, adopting UHC and how it is implemented are primarily political choices (10). Since medicalizing health props up an apolitical position with regard to UHC it stands to reason that challenging medicalization could be served by introducing the importance of political processes to the realisation of UHC. This then requires political debates and commitments to equity, quality, and collective responsibility that could fulfil the expectations of UHC to provide a significant global health transition and ‘transform poverty, hunger, and disease’. Furthermore, determining what individuals or communities want from health-care coverage are not technical issues either, but ones of social value (8). Finally, more evidence is clearly needed on the outcomes and implications of UHC in practice, particularly in regard to health goals that aim for equitable and quality care.
